# cMyBP-C in hypertrophic cardiomyopathy: gene therapy and small-molecule innovations

**DOI:** 10.3389/fcvm.2025.1550649

**Published:** 2025-02-26

**Authors:** Patrick T. Wood, Morgan M. Seffrood, Brett A. Colson, Julian E. Stelzer

**Affiliations:** ^1^ Department of Physiology and Biophysics, School of Medicine, Case Western Reserve University, Cleveland, OH, United States; ^2^ Department of Cellular & Molecular Medicine, University of Arizona, Tucson, AZ, United States

**Keywords:** hypertrophic cardiomyopathy, cMyBP-C, myosin binding protein C, gene therapy, AAV9 gene transfer, small-molecule, high-throughput screening, small molecule therapy

## Abstract

Hypertrophic cardiomyopathy (HCM) is a genetic disorder in the heart caused by variants in sarcomeric proteins that disrupt myocardial function, leading to hypercontractility, hypertrophy, and fibrosis. Optimal cardiac function relies on the precise coordination of thin and thick filament proteins that control the timing, magnitude of cellular force generation and relaxation, and *in vivo* systolic and diastolic function. Sarcomeric proteins, such as cardiac myosin binding protein C (cMyBP-C) play a crucial role in myocardial contractile function by modulating actomyosin interactions. Genetic variants in cMyBP-C are a frequent cause of HCM, highlighting its importance in cardiac health. This review explores the molecular mechanisms underpinning HCM and the rapidly advancing field of HCM translational research, including gene therapy and small-molecule interventions targeting sarcomere function. We will highlight novel approaches, including gene therapy using recombinant AAV vectors and small-molecule drugs targeting sarcomere function.

## 1 Introduction

Heart disease is a major health challenge in the United States, contributing to millions of hospitalizations and accounts for 1 in every 5 deaths annually (1). Among the various forms of heart disease, hypertrophic cardiomyopathy (HCM) is the most common genetic cardiomyopathy, affecting approximately 1 in 500 people in the U.S (2). GWAS studies have shown that HCM is an autosomal dominant disease caused by one or more genetic variants in sarcomeric proteins (3–7). Alterations to sarcomere proteins result in either a gain or loss of function at the cellular level. This leads to dysregulation in the precise coordination of cardiac contraction and relaxation at the whole organ level.

Clinically, HCM presents as a heterogeneous phenotype that leads to variable degrees of cardiac remodeling (8–10). Common features of HCM include asymmetrical thickening of the left ventricular (LV) wall, hyperdynamic systolic performance, and a preserved or elevated left ventricular ejection fraction (LVEF) (10, 11). HCM is defined as either non-obstructive (nHCM), or obstructive (oHCM) depending on the severity of hypertrophy and the degree of left ventricular outflow tract obstruction (LVOTO), with the majority of patients with HCM experiencing LVOTO either at rest or during movement (10, 12).

Historically, therapeutic interventions such as beta-blockers (b-blockers), and non-dihydropyridine calcium channel blockers (CCBs) have been used to treat HCM symptoms by modulating adrenergic and calcium signaling pathways to improve patient quality of life (10, 13), but these therapies do not directly address the underlying problem of pathogenic variants in sarcomeric proteins (10, 14). Recent advances in therapeutic interventions are shifting toward directly targeting the primary defects found in altered sarcomere proteins to restore normal cardiac function at the molecular level (13, 15). This emerging paradigm highlights the growing interest in linking genotype to phenotype and connecting basic research to clinical practice.

## 2 cMyBP-C regulates contraction

Cardiac sarcomeric function is governed by the complex interaction of actin and myosin, in addition to several structural/regulatory proteins, such as the troponin complex, tropomyosin, and cardiac myosin binding protein C (cMyBP-C). These proteins are highly regulated and modulate contractile function in response to extracellular signaling (16). Numerous clinical studies demonstrate that pathogenic genetic variants of the myosin *MYH7* gene or cMyBP-C *MYBPC3* gene account for roughly 80% of all genetic variants that result in HCM (3, 9, 17), with *MYBPC3* genetic variants being the most common (3, 18, 19).

Variants of *MYBPC3* are a growing area of research due to its regulatory role in the sarcomere [see reviews, (19, 20)]. Localized in the C-zone of the A-band, cMyBP-C is anchored to the thick filament and arranged to create nine regularly spaced stripes ∼42 nm apart (21, 22). The 140-kDa protein consists of 11 globular immunoglobulin and fibronectin-like domains (C0–C10), with a flexible and phosphorylatable M-domain between C1 and C2, a key regulatory region (23–25).

Under the control of β-adrenergic stimulation, cMyBP-C's phosphorylation is regulated to control pressure development and relaxation *in vivo* (26). Unphosphorylated, cMyBP-C preferentially binds to myosin and acts as a mechanical tether that restrains the myosin heads of the thick filament (27–29). In this state, the myosin heads are spatially restricted, reducing their mobility and probability of interacting with actin, thus slowing the rate of force generation (20, 26, 30). The sequestration of the myosin heads is relieved via phosphorylation of the N-terminal domains (NTDs) of cMyBP-C, specifically at sites within the M-domain (20, 23, 31). The phosphorylation sites are targeted by various kinases, including protein kinase A (PKA), Ca2+/calmodulin-dependent protein kinase II, protein kinase C (PKC), and protein kinase G (PKG) allowing for precise adrenergic control over cardiac contraction (23, 32–34). In response to exercise or stress where increased cardiac output is necessary, phosphorylation of cMyBP-C relieves the restriction of myosin and reduces the tethering of cross-bridges via a reduction in cMyBP-C affinity for both myosin subfragment 1 and 2 (S1, S2) and actin (25, 35–37). The reduced constraint on myosin increases the probability of cross-bridge formation and enhances the transition of cross-bridges to force-bearing states, thereby accelerating contraction and increasing force generation (20, 26, 38, 39).

*In vitro* studies have also shown that specific regions of cMyBP-C, particularly the C0-C2 region, also modulate force generation (36, 40–42) by binding to the thin filament at low calcium concentrations to displace tropomyosin, and activate the thin filament (40, 43, 44). This way through interactions with myosin, actin, or both, cMyBP-C provides a high level of fine-tuning to cardiac contractility with a relatively low cMyBP-C to myosin ratio (1:8) and cMyBP-C to actin ratio (1:12) (36, 45).

## 3 Pathogenic variants of *MYBPC3* lead to the development of HCM

**Figure 1:**
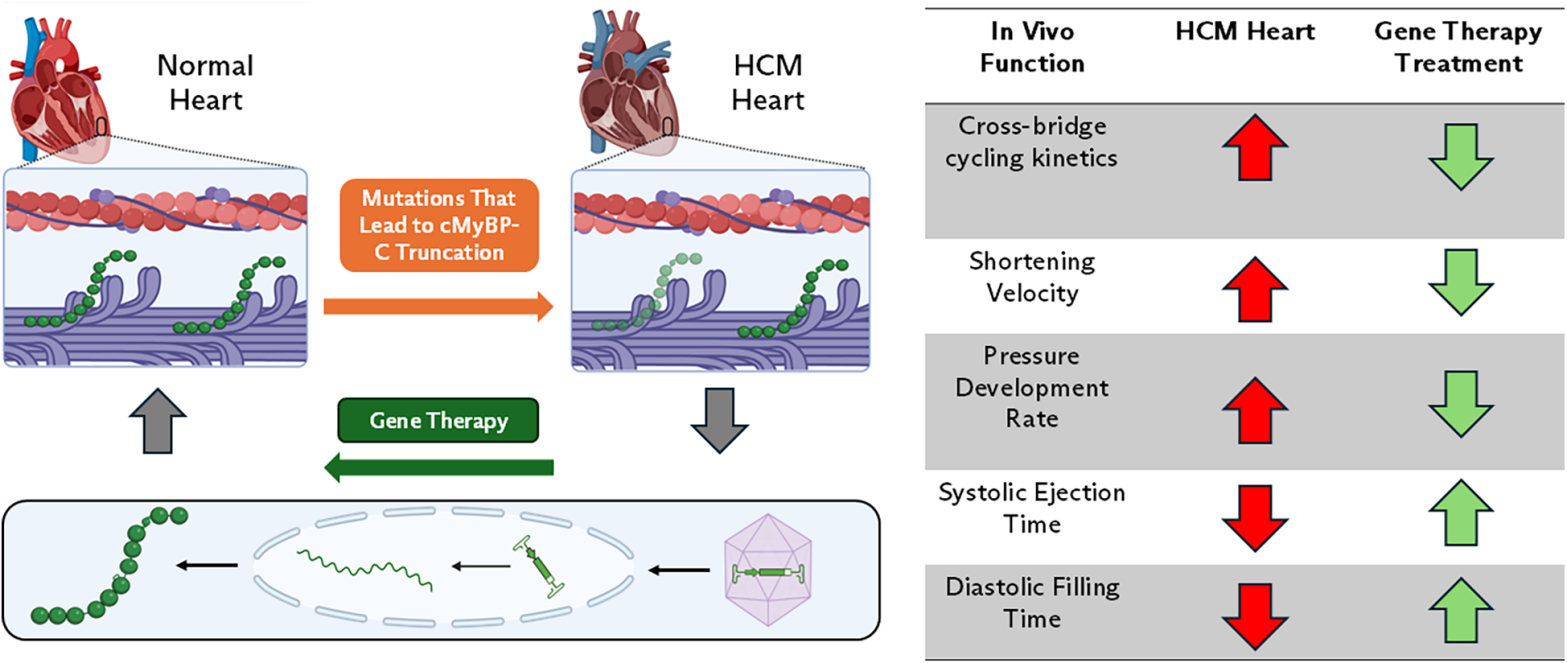
cMyBP-C haploinsufficiency-based HCM. In this form of disease, genetic variations in *MYBPC3* result in prematurely truncated cMyBP-C protein. Reduction in cMyBP-C expression disrupts the stoichiometric balance between cMyBP-C, myosin, and actin in the sarcomere resulting in dysregulation of sarcomere contractility. Gene therapy using AAV9 aims to restore cMyBP-C levels to normal and mechanistically slow shortening velocity and increase thin filament drag. *In vivo*, this normalizes rates of pressure development and systolic ejection times, thereby improving systolic ejection and diastolic filling.

In patients with *MYBPC3-*based HCM, the well-regulated control of the sarcomere is lost due to genetic variants in the protein. Pathogenic variants in *MYBPC3* occur primarily in two forms: nonsense and missense, both of which play distinct roles in the development of HCM (9). The majority of pathogenic HCM-causing *MYBPC3* variants are heterozygous frameshift, nonsense, or splice site variants (46, 47). These genetic variants result in the introduction of premature termination codons in one allele and theoretically result in the production of truncated cMyBP-C protein, however, truncated cMyBP-C proteins have not been observed in patient HCM cardiac tissue (47–49). Data from patient-derived, *MYBPC3*-variant induced pluripotent stem cell cardiomyocytes (iPSCMs) suggests that the *MYBPC3* mRNA transcript is degraded through nonsense-mediated RNA decay resulting in protein haploinsufficiency (50). Reduction in cMyBP-C protein expression disrupts the stoichiometric balance between cMyBP-C, myosin, and actin in the sarcomere, leading to a loss of regulation over cross-bridge formation, and force generation (Figure 1) (19, 29, 48, 51). Patients with heterozygous *MYBPC3* truncating variants can have a range of symptoms that develop over their life span, while patients with homozygous *MYBPC3* truncating variants develop severe HF within the first year of life (52–54).

**Figure 2:**
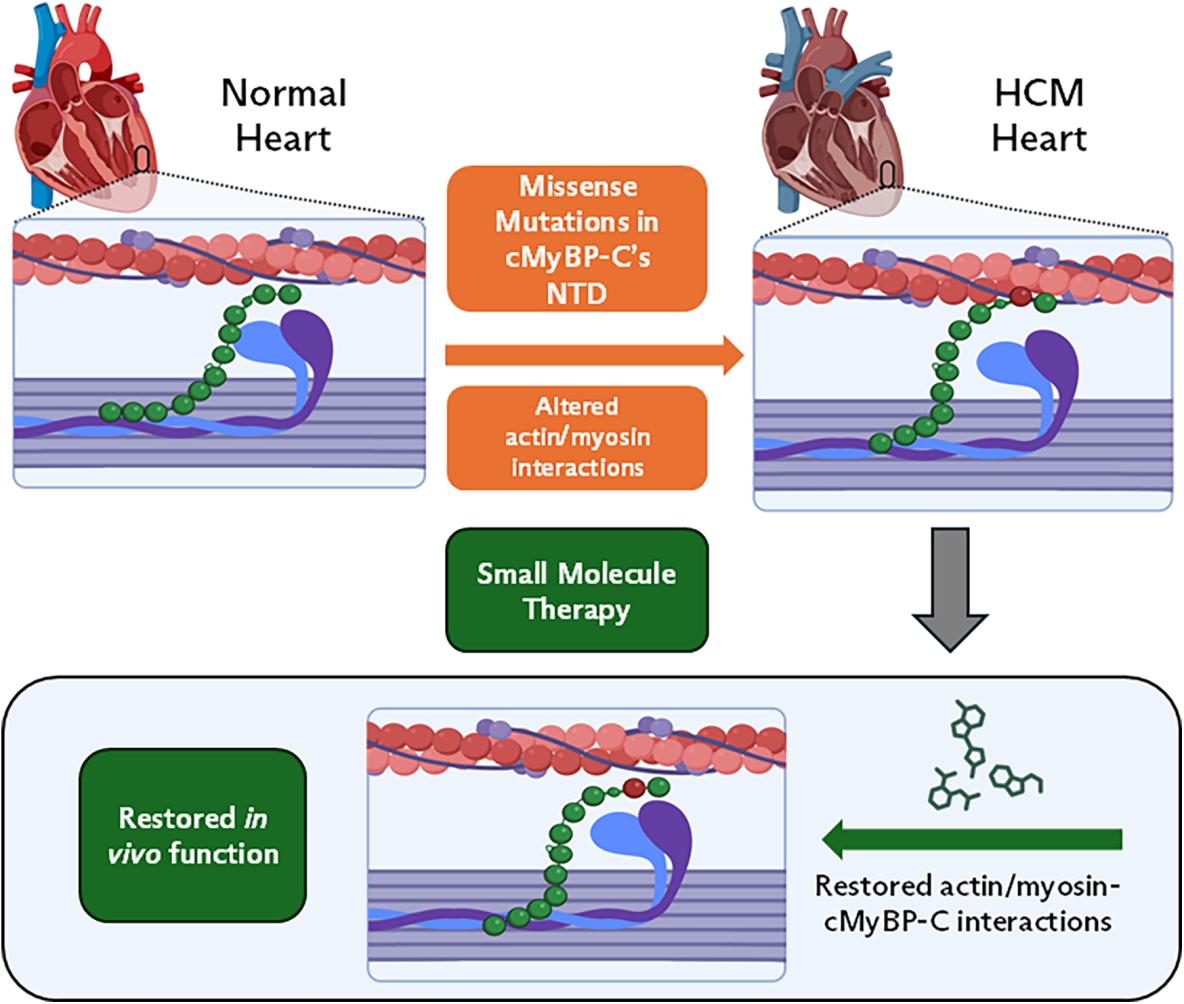
cMyBP-C missense variations in HCM. In this form of disease, genetic variations in *MYBPC3* result in single nucleotide substitutions. These variations can have a wide range of effects on cMyBP-C's ability to regulate actin/myosin interactions. Small molecule (SM) therapy can revert hyper-contractility to normal, by restoring cMyBP-C's interactions with actin/myosin. This will have similar effects to *in vivo* function seen in Figure 1 table inset (right).

In contrast, pathogenic missense variants in *MYBPC3* involve a single nucleotide substitution that results in the replacement of one amino acid with another in the protein (19, 55). Unlike truncating variants that result in haploinsufficiency, pathogenic missense variants typically lead to the production of full-length proteins that can be incorporated into the sarcomere, resulting in a dominant negative effect (19, 55). These variants impair cMyBP-C's ability to regulate actin/myosin interactions, however, the exact mechanism of impairment may vary depending on where in the protein the genetic variation is located (32, 56, 57). Some variants may alter cMyBP-C's structure to increase/decrease actin/myosin binding while others may affect how the protein is phosphorylated (32, 57–61). The example in Figure 2 highlights how a theoretical variation in the NTD can cause a change in protein structure, leading to increased cMyBP-C/actin binding and decreased cMyBP-C/myosin binding. Due to the low population frequency of missense variants, it is difficult to interpret how patients may develop disease and respond to treatments. This has led to a rise in novel molecular and computational screens that can be used to improve individualized patient care (58, 59, 62).

## 4 Emerging treatments for heart failure and HCM

Treating HCM is difficult, in part, due to disease heterogeneity and long patient histories with several potential confounding variables. Current treatment strategies focus on relieving symptoms of HCM and improving patient quality of life through the use of pharmaceuticals with negative inotropic and chronotropic effects (10). Current AHA/ACC guidelines for the treatment of HCM suggest using non-vasodilating b-blockers first, followed by non-dihydropyridine CCBs (10). At the molecular level, b-blockers and CBCs decrease systolic function by blocking signaling pathways, inhibiting PKA, and decreasing intracellular calcium levels in cardiac myocytes to reduce contractility (63, 64). Due to their indirect mechanism of action, these drugs have several off-target side effects and do not specifically address genetic variants in sarcomeric proteins that a patient may have (65, 66).

Recent therapeutic advances are changing the paradigm in the treatment of HCM. The concept of direct modulation of the sarcomere provides an alternative approach to minimize off-target effects associated with indirect treatments (13, 15, 65). Unlike therapies that focus on symptom relief, direct sarcomere modulation addresses the regulation of contraction at the mechanistic level to reduce hypercontractility. Current direct modulation therapies for HCM treatment involve selective, allosteric, small molecules that specifically modulate cardiac myosin to directly affect the probability of actin-myosin cross-bridge formation (67–70). Drugs such as Mavacamten (MYK-461) and Aficamten (CK-274) are currently undergoing clinical trials to explore their effectiveness in treating and managing HCM (67, 68, 71). These novel therapeutics are first in class for the treatment of HCM, but like any new therapy, they have their caveats.

Clinical trials for Mavacamten, PIONEER-HCM, EXPLORER-HCM, and VALOR-HCM, were conducted on a limited patient population with oHCM (66, 71–73). These patients did not undergo genetic screening; leaving uncertainties about how Mavacamten affects patients with *MYBPC3* genetic variants or other genetic variants. Studies using myocardium isolated from cMyBP-C^−/−^ knockout (KO) mice, noted that higher concentrations of MYK-461 were required to achieve similar degrees of force depression compared with WT myocardium (74) suggesting genetic variants may play a role in the efficacy of MYK-461. Furthermore, it remains unclear how patients with other types of HCM, such as non-obstructive HCM (nHCM), may respond to Mavacamten treatment. These limitations ultimately reduce the utility of this drug to a smaller subset of the population. To address the critical need for the development of new therapies, there are currently two options under investigation: gene therapy or small molecule therapy (62, 75–80). Both therapy options allow for a more personalized and variant-specific approach to treatment.

## 5 Gene therapy for *MYBPC3* variants

The goal of gene therapy is to correct sarcomeric variants that drive the development of HCM by correcting haploinsufficiency, engineering therapeutic proteins, or modifying the expression of existing genes (81). There are several modalities of gene therapy for treating genetic-based HCM, including gene replacement, silencing, or editing.

For patients with truncation variants of *MYBPC3,* treatments that involve increasing cMyBP-C protein expression have shown promising results in small animal studies (82). Gene replacement therapies use cardiac targeting expression vectors, such as AAV9 vectors, with cardiac-specific promotors to increase the amount of cMyBP-C protein in the cardiomyocyte. This can be done with full-length peptides or therapeutic cMyBP-C protein fragments (Figure 1) (75, 77, 78, 83). Physiologically, *in vivo* reintroduction of cMyBP-C protein has been shown to slow shortening velocity in cMyBP-C ^−/−^ mice (83). At the molecular level, increasing the amount of cMyBP-C protein or protein fragments restricts myosin head availability, leading to the normalization of cross-bridge kinetics and cycling (75, 77, 83). Additionally, the reintroduction of protein or fragments has been shown to restore cMyBP-C phosphorylation via β-adrenergic stimulation (77). Restoration of cMyBP-C at the molecular level translates *in vivo* to a normalized rate of pressure development and systolic ejection time, thereby improving systolic ejection and diastolic filling (75, 77, 83).

The first *MYBPC3*-based clinical trial to treat HCM patients using a gene replacement technique is underway. The MyPeak-1 (Phase 1b) clinical trial started in 2023 to evaluate the safety and clinical efficacy of an AAV gene replacement therapy “TN-201” for pathogenic *MYBPC3* variants related to HCM (NCT05836259). The therapy is a one-time treatment using an IV infusion of full-length *MYBPC3* AAV. To be included in the trial, patients must be diagnosed with HCM (oHCM or nHCM) and have a LVEF >45%. Additionally, patients must undergo genetic testing to confirm the presence of pathogenic or likely pathogenic *MYBPC3* truncating variants. During the 5-year study, clinicians will monitor changes in patient RV cMyBP-C protein levels, VO_2_-max, LV mass index, LV filling pressure, and New York Heart Association functional class changes (80). The goal is to halt disease progression or reverse symptoms over 5 years. Interim data from Cohort-1 showed that a low dose of TN-201 was well tolerated and the trial is currently recruiting patients for a higher dose trial, however, further public data is limited at the time of writing (80).

Other therapeutic options may be better suited for patients with missense variants that result in full-length proteins with negative effects. Under these conditions, gene replacement therapy might not fully correct the HCM phenotype as the missense variant is still transcribed. To correct genetic variants that result in cMyBP-C structural changes causing hypercontractility, gene silencing techniques could use rAAV to deliver small interfering RNAs (siRNAs) to stop transcription, or silence, pathogenic genes (84). This technique successfully prevented HCM pathology for at least 6 months in mice carrying a pathogenic variant in the β-myosin heavy chain gene (76) and could be adapted for *MYBPC3* variants. However, SiRNA therapy could be difficult to implement in *MYBPC3-*HCM patients, as this therapy could result in a haploinsufficient phenotype.

Alternatively, missense variants can be corrected or edited using a genome editing technique instead of silenced. By delivering an RNA-guided Cas9 nuclease via AAV9, the variant gene can be edited back to its non-variant state (79). Genetic editing of β-myosin heavy chain gene R403Q variant mice corrected 70% of variant transcripts and prevented the associated HCM phenotype (79). While promising, gene therapy is still a novel technique and faces barriers like manufacturing ability, scalability, and cost (85). Additionally, some patients may also have strong immunity to AAV-based therapies that prevent delivery and transcription of the treatment, thus these patients may require higher doses (85) or alternative therapies, such as small molecules.

## 6 High-throughput screening for novel small molecules

Small-molecule drugs that bind to cMyBP-C and alter its interaction with the thick and thin filaments could be used to improve cardiac function in HCM patients with genetic variants of *MYBPC3*. By acting to inhibit or enhance cMyBP-C binding with actin and myosin, small molecules directly target the dysfunctional protein interactions at the sarcomeric level to normalize cardiac muscle performance at the whole heart level (Figure 2).

To treat HCM patients with hyper-contractility and incomplete relaxation, the mechanisms of drug action on cMyBP-C function can either increase or decrease interactions with actin and myosin to result in reduced contractility and enhanced relaxation. In one scenario, several HCM-linked missense variants located in the M-domain of cMyBP-C exhibit enhanced interactions with actin and myosin (30, 61). Thus, compounds designed to modulate actin-cMyBP-C interactions and reduce myocardial ATPase activity (58, 62) could result in therapies that directly normalize affinities and conformations of variant cMyBP-C with the myofilaments, improving heart function. Contrary, in the case of cMyBP-C haploinsufficiency (i.e., less cMyBP-C protein) or missense variants in non-cMyBP-C sarcomeric proteins (i.e., beta-myosin) that result in hyper-contractility due to an increased probability of cross-bridges, drugs designed to modulate cMyBP-C interactions with myosin to decrease actin-myosin interactions in favor of relaxed myocardium and reduced contractility (27–29, 86) could mitigate hyper-contractility in these patients. Alternatively, drugs that target cMyBP-C to modulate its binding to actin and tropomyosin to lessen cMyBP-C-induced thin filament activation that sustains force generation (43, 44) could reduce hyper-contractility and enhance relaxation. Thus, cMyBP-C targeting modulators for the treatment of HCM would be expected to impact *in vivo* cardiac performance by suppressing systolic contractility while enhancing diastolic relaxation to ameliorate symptoms.

There are currently no known cMyBP-C binding drugs despite the desirability of such modulators of cMyBP-C. This is due to the lack of high-throughput screening (HTS) assays to identify drugs that bind specifically to cMyBP-C and alter its function. Current industry screening methods mostly rely on myosin ATPase assays which have been used to identify small molecule treatments for HCM such as Mavacamten and Omecamtiv mecarbil (69, 87) which target cardiac myosin. It is possible that these myosin ATPase assays (activated by actin, Ca^2+^, cMyBP-C NTDs), could be used with the addition of cMyBP-C or its fragments (e.g., C0-C2); however, these methods are not ideally suited for identifying cMyBP-C-specific binding compounds (without actin or myosin binding properties). Other approaches such as using genetically altered or iPSC-derived cardiomyocytes in contractility assays, are time-consuming, expensive, and labor-intensive and still have their caveats. Testing for small molecules that specifically target and bind to cMyBP-C and modulate its interactions with actin or myosin is the critical first step in the drug development process. Using HTS assays expedites this process and accelerates the identification of drugs for treating HCM.

Fluorescence lifetime (FLT) and FLT-detected FRET assays using probes on human cMyBP-C C0-C2, and actin (or myosin) have recently been developed (58, 60–62). Combined with a plate reader, drug screens of ∼1,300–2,800 compounds have been performed (58, 62). Pharmacologically active compounds identified with changes in lifetime or FRET indicated a change in cMyBP-C binding to actin. Further testing of some of the compounds identified from the HTS *in vitro* assays on function revealed effects on modulating myosin ATPase activity measured by *in situ* assays using skinned cardiac or skeletal muscle (88). Thus, the HTS assays developed can quickly and accurately detect valid cMyBP-C binding compounds to be further validated in downstream functional assays of muscle function (62).

Currently, small-molecule modulators binding to C0-C2 have been identified to either inhibit or enhance interactions with actin (58, 62). A subset of these modulators affect the Ca^2+^-sensitivity ATPase activity in cardiac myofibrils (62). For example, Pneumocandin B_0_ increased ATPase activity in cardiac myofibrils and was identified to increase FRET between cMyBP-C C0-C2 and actin in the screen (62), suggesting it may be an activator of cMyBP-C. In another example, Enoxaparin sodium decreased ATPase activity in cardiac myofibrils and was identified to decrease FRET between C0-C2 and actin in the screen (62), suggesting it could be an inhibitor of cMyBP-C. A cMyBP-C C0-C2-myosin assay has also been developed (61). In addition to primary HTS for compounds affecting myosin-cMyBP-C interactions, these assays are also useful for deprioritizing actin and myosin-binding drugs (to prioritize cMyBP-C binding drugs). Future work will establish the efficacy of screened compounds *in vitro* and *in vivo* models of HCM.

Ideal compounds would only bind to cardiac MyBP-C and not skeletal MyBP-C (62). Additionally, they may also bind to the interface of cMyBP-C in complex with actin or myosin. The drugs should have good ADMET properties, such as cell permeability, be nontoxic, and have minimal side effects. Additionally, ideal compounds should effectively treat certain HCM subtypes (obstructive or non-obstructive, cMyBP-C or non-cMyBP-C variants).

## 7 Future directions

Several avenues for future investigation are in development using the techniques described above to treat HCM and other cardiomyopathies. Gene therapies could introduce designer cMyBP-C proteins that mimic different phosphorylation states, thereby providing more nuanced and targeted treatment for different cardiomyopathies (75). The engineered cMyBP-C variants would be designed to express phospho-mimetic variants that can modulate contractility in specific HCM subtypes or other cardiomyopathies where increased or decreased contractility might be beneficial. Similarly, drugs that mimic cMyBP-C phosphorylation by affecting cMyBP-C structure (30, 60, 62, 89) and function (90, 91) can enhance myocardial contraction and relaxation. Importantly, these perturbations would be independent of adrenergic stimuli or neurohormonal signaling, which are often dysregulated in heart failure.

cMyBP-C-targeted small molecules could also treat other cardiac disorders, such as dilated cardiomyopathy (DCM) by reducing myosin and increasing actin interactions to enhance contractility. Likewise, non-genetic HF (HFrEF/HFpEF) by similar mechanisms of action to change cMyBP-C interactions as in HCM/DCM for alleviating dysfunction of hypo- or hyper-contractility could provide effective new intervening therapies.

HCM is a diverse and complicated disease that can have multiple genetic aspects in different sarcomere and potentially non-sarcomere proteins (6, 7). By working closely with clinicians and developing genetic screens and patient-specific treatment regimes, it may be possible to combine multiple therapeutic approaches to deliver a personalized approach to HCM treatment. Additionally, investigating the safety and efficacy profiles of these novel interventions in pediatric populations may offer unprecedented opportunities for preventing or delaying the onset of HCM in genetically susceptible individuals (52). These novel therapies will need to be tested against current standard-of-care treatments to establish cMyBP-C-targeted therapies within the broader therapeutic landscape.
